# Immunogenicity and safety of primary three-dose series with diphtheria, tetanus and pertussis (acellular, three components) combined vaccine, adsorbed in 3 months infants

**DOI:** 10.3389/fimmu.2026.1874647

**Published:** 2026-07-07

**Authors:** Wei Zhang, Chen Wei, Haitao Huang, Xue Wang, Zhiqiang Xie, Yan Wu, Feiyu Wang, Lili Huang, Xuewen Wang, Xiuwen Sui, Peng Wan, Zhe Chao, Jinbo Gou, Bozhi Shi, Jia Liu, Tao Zhu, Liyong Yuan, Yanxia Wang

**Affiliations:** 1 Henan Provincial Center for Disease Control and Prevention, Henan Key Laboratory of Infectious Pathogenic Microbiology, Zhengzhou, China; 2 National Institutes of Food and Drug Control, Beijing, China; 3 State Key Laboratory of Drug Regulatory Science, Beijing, China; 4 CanSino Biologics Inc., Tianjin, China; 5 Shanghai Imstat Medical Technology CO., LTD, Shanghai, China

**Keywords:** clinical trial, co-purified DTaP, DTcP, immunogenicity, safety

## Abstract

This study aimed to evaluate the safety and immunogenicity of a novel diphtheria, tetanus, and pertussis (acellular, three components) combined vaccine, adsorbed (DTcP) vaccine compared to the existing co-purified DTaP vaccine in China. In this partially randomized, blinded, controlled Phase III trial, we assessed a DTcP vaccine against two controls: a licensed co-purified DTaP and a DTaP-IPV-Hib vaccine. A total of 1,140 infants (3 months old) received a 3-dose primary series at 3, 4, and 5 months. Both of DTcP and co-purified DTaP had lower incidences of overall adverse reactions than the DTaP-IPV-Hib vaccine (17.99%/18.08% vs. 21.96%). The DTcP vaccine demonstrated non-inferiority and superiority over the co-purified DTaP for anti-PT and anti-FHA immunogenicity. In addition, the DTcP achieved a 98.16% seroconversion rate for anti-FHA, versus only 14.29% for the co-purified DTaP. Besides, DTcP vaccine also induced high antibody level in GMC results. Specifically, the anti-PT GMC was 85.10 in the DTcP group versus 49.86 in the DTaP group (*P* < 0.001) and 74.25 in the DTaP-IPV-Hib (*P* = 0.001). For anti-FHA, the GMC was 108.28 in the DTcP group compared to only 10.69 in the DTaP group. In conclusion, DTcP vaccine induces a significantly more robust immune response to key pertussis antigens --compared to the co-purified DTaP, with a comparable safety profile. These data support the potential optimization of China’s immunization strategy by transitioning from co-purified to component-based acellular pertussis vaccines to address waning immunity and the ongoing pertussis resurgence.

**Clinical trial registration:**

## Introduction

Pertussis, caused by *Bordetella pertussis*, remains a major cause of infant mortality and morbidity globally. World Health Organization has highlighted its significant impact on children under 5 years of age, particularly in low- and middle-income countries (1). The epidemiology of pertussis in China has undergone a dramatic and concerning transformation in the post-COVID-19 pandemic period, characterized by an unprecedented resurgence in incidence and a fundamental shift in the affected demographic. China witnessed an alarming surge of pertussis cases in 2024, with reported cases rising to 476,690 and 31 fatalities. This represents a more than 12-fold increase from the 38,205 cases recorded in 2023 and signifies the largest outbreak in decades (2, 3). This dramatic surge follows a period of relative stability, where reported cases remained at sustained levels around 30,000 annually, as seen with the 30,027 cases in 2019 through the 2020–2021 period (4). Epidemiological data from Europe and the United States highlight the burden of pertussis among infants, who face the highest risk of severe outcomes. In Europe, the incidence of pertussis under 1 year of age is 73.6 per 100,000, with hospitalization rates reaching 63.1% for those under 3 months and ranging from 30.3% to 61.4% for infants aged 3–5 months (5). China’s planned immunization program, initiated in 1978, initially utilized whole-cell pertussis vaccines, leading to a significant decline in pertussis incidence. A major policy shift occurred from 2007 onwards, with the national program transitioning to co-purified acellular pertussis (aP) vaccines, specifically the diphtheria, tetanus, and acellular pertussis vaccine (DTaP), due to its favorable reactogenicity profile (6). However, accumulating evidence suggests that aP vaccines do not always confer equivalent long-term protection. Vaccines manufactured using traditional co-purification methods, which is a process in which multiple antigenic components are co-purified from *Bordetella pertussis* cultures in a single step, may yield a less defined antigenic mixture with variable composition, potentially compromising immunogenicity and protection durability (7). These co-purified pertussis antigen complexes are then proportioned and mixed with separately prepared diphtheria toxoid and tetanus toxoid to form the final vaccine (7). Additionally, co-purified vaccines may face challenges in standardizing antigen composition across batches and characterizing each component’s individual contribution to efficacy and safety (8).

This study evaluated the safety and immunogenicity of a novel diphtheria, tetanus and pertussis (acellular, three components) combined vaccine, adsorbed (DTcP) in 3-month-old infants. The experimental DTcP, which utilizes a novel gene expression-based platform for antigen purification and detoxification, was compared against licensed DTaP-IPV-Hib and co-purified DTaP vaccine currently used in China for infants at 3 months of age. DTcP has since been approved for marketing in China under the trade name Tripecia^®^. As the first domestically developed DTcP to complete standardized clinical trials under a (3, 4, 5 months) primary immunization schedule, this study represented the first positive-controlled Phase III clinical trial against a co-purified DTaP vaccine, thereby generating critical evidence to support the optimization and potential upgrading of national pertussis immunization strategies.

## Methods

### Study design and participants

This was a partially randomized, partially blinded, controlled trial conducted in Henan Province, China. The study recruited healthy 3-month-old infants (90–119 days) who had not previously received any diphtheria, tetanus, pertussis, IPV, Hib, or pneumococcal conjugate vaccines. The exclusion criteria are described in the **Supplementary Material**. Written informed consent was obtained from parents or legal guardians before enrollment. The protocol was approved by the ethics committee of Henan Province Center for Disease Control and Prevention and was performed in accordance with the Declaration of Helsinki. The study was registered on ClinicalTrial.gov (NCT05951725).

### Randomization and blinding

Random numbers for 3-month-old participants were generated using block randomization. Participants were randomly assigned to the DTcP group and the DTaP group in a 1:1 ratio. The DTaP-IPV-Hib group was not randomized (open-label). An additional 380 participants were assigned to receive DTaP-IPV-Hib. All participants completed a three-dose primary vaccination series at 3, 4, and 5 months of age in accordance with China’s Expanded Program on Immunization (EPI) schedule.

To maintain blinding between the DTcP and DTaP groups (which had identical outer packaging but different inner packaging), vaccine preparation personnel were separated from vaccination and outcome assessment personnel. Vaccine administrators did not participate in any safety follow-up or immunogenicity assessments. For immunogenicity endpoints, all serum samples from the three groups were tested in parallel with laboratory personnel blinded to group allocation.

The primary immunogenicity endpoints were the non-inferiority and superiority of seroconversion rates and geometric mean concentrations (GMCs) of anti-pertussis toxoid (PT), filamentous hemagglutinin (FHA), pertactin (PRN), diphtheria toxoid (DT), tetanus toxoid (TT), antibodies 30 days after primary vaccination. Geometric mean increases (GMIs) and seropositivity rate of PT, FHA, PRN, DT, TT antibodies 30 days after the primary vaccination were the secondary objectives. The primary safety endpoints were the incidence of adverse reactions within 30 days after primary vaccination.

### Laboratory testing

Antibody responses against PT, FHA, PRN, DT, and TT were quantitatively measured using a multiplex Luminex-based immunoassay. Coating antigens were: native proteins for PT, FHA, and PRN; and toxoids for DT and TT. Lower limits of quantification (LLOQ) differed accordingly: for PRN, LLOQ was 0.10 IU/mL pre-vaccination and 3.8 IU/mL post-vaccination. For GMC calculations, values below the LLOQ were assigned half of the LLOQ. Standard curves were generated using nine 2-fold serial dilutions of reference serum. Quality control sera were tested with a coefficient of variation requirement of <20%. A five-parameter logistic regression was used for calculation. The detailed methodology was performed as previously described (9).

### Vaccines

DTcP (experimental vaccine) was produced by CanSino Biologics Inc., which was developed by modifying the existing pertussis vaccine production strain (CMCC58003). Three genetically engineered pertussis strains expressing FHA, PT, and PRN were constructed, followed by comprehensive clone screening with verification of both genotype and phenotype. The final vaccine formulation was prepared by mixing five bulk antigens (FHA, PT, PRN, DT, and TT) in appropriate proportions. Each 0.5 mL single dose contained: 25 μg of FHA, 25 μg of PT, 8 μg of PRN, 12.5 Lf of DT, and 3.5 Lf of TT. A dual-adjuvant system (aluminum hydroxide and aluminum phosphate) was employed, with a total aluminum content of 0.24 mg per 0.5 mL dose. The adsorption process was performed under controlled pH and temperature conditions. DTaP (co-purified control vaccine) was produced by Wuhan Institute of Biological Products Co., LTD. It is formulated from acellular pertussis vaccine stock solution, DT, and TT combined with aluminum hydroxide adjuvant. It contains thimerosal as a preservative. Each 0.5 mL dose contains a pertussis vaccine potency of not less than 4.0 IU (total pertussis activity; individual PT/FHA/PRN quantities in μg are not available due to the co-purification process), a diphtheria potency of not less than 30 IU, and a tetanus potency of not less than 40 IU. DTaP-IPV-Hib (component control vaccine) was produced by Sanofi Pasteur. Each 0.5 mL dose contained diphtheria toxoid, tetanus toxoid, PT, FHA, IPV and Hib antigens. PRN antigen is absent from this vaccine. All the experimental and control vaccines were administered via an intramuscular injection into the anterolateral thigh.

### Statistical analysis

Sample size was calculated based on estimated anti-PRN antibody seroconversion rate. Using the single-proportion estimation feature (Tests for One Proportion) in NCSS-PASS (Version 16.0), with a confidence level of 1-α = 0.95 (two-tailed), assuming the seroconversion rate for the lower PT and FHA components in the experimental vaccine is approximately 0.9 (not less than 80%), H_0_ is approximately 0.72, and the overall power is set to 1-β = 0.9, Assuming a post-vaccination PRN antibody seroconversion rate of no less than 0.8, the calculated required sample size is 304 participants. Accounting for a 20% dropout rate, each group requires 380 participants.

Statistical analyses in this study were performed using SAS 9.4 or higher (SAS INSTITUTE INC, USA) software programming. All immunogenicity analyses were performed on the Per-Protocol Set, defined as participants who completed all three doses, had no major protocol deviations, and had evaluable serum samples at both baseline and day 30. Seroconversion was defined as follows: for anti-DT and anti-TT, post-vaccination concentration ≥0.1 IU/mL (if baseline <0.1) or a fourfold increase (if baseline ≥0.1). For anti-PT, anti-FHA, and anti-PRN, post-vaccination concentration ≥20 IU/mL (if baseline <5) or a fourfold increase (if baseline ≥5 to <20, or ≥20). Seropositivity thresholds were: anti-PT and anti-FHA ≥20 IU/mL, anti-DT and anti-TT ≥0.01 IU/mL, and anti-PRN ≥5 IU/mL. Two-tailed tests were used throughout, with P ≤ 0.05 considered statistically significant. For non-inferiority comparisons of seroconversion rate differences, the non-inferiority margin was -10% (lower bound of 95% CI > -0.10), and superiority was declared if the lower bound exceeded 0. For GMC ratios, the non-inferiority margin was 0.67 (lower bound of 95% CI ≥0.67), and superiority was declared if the lower bound exceeded 1.

## Results

**Figure 1:**
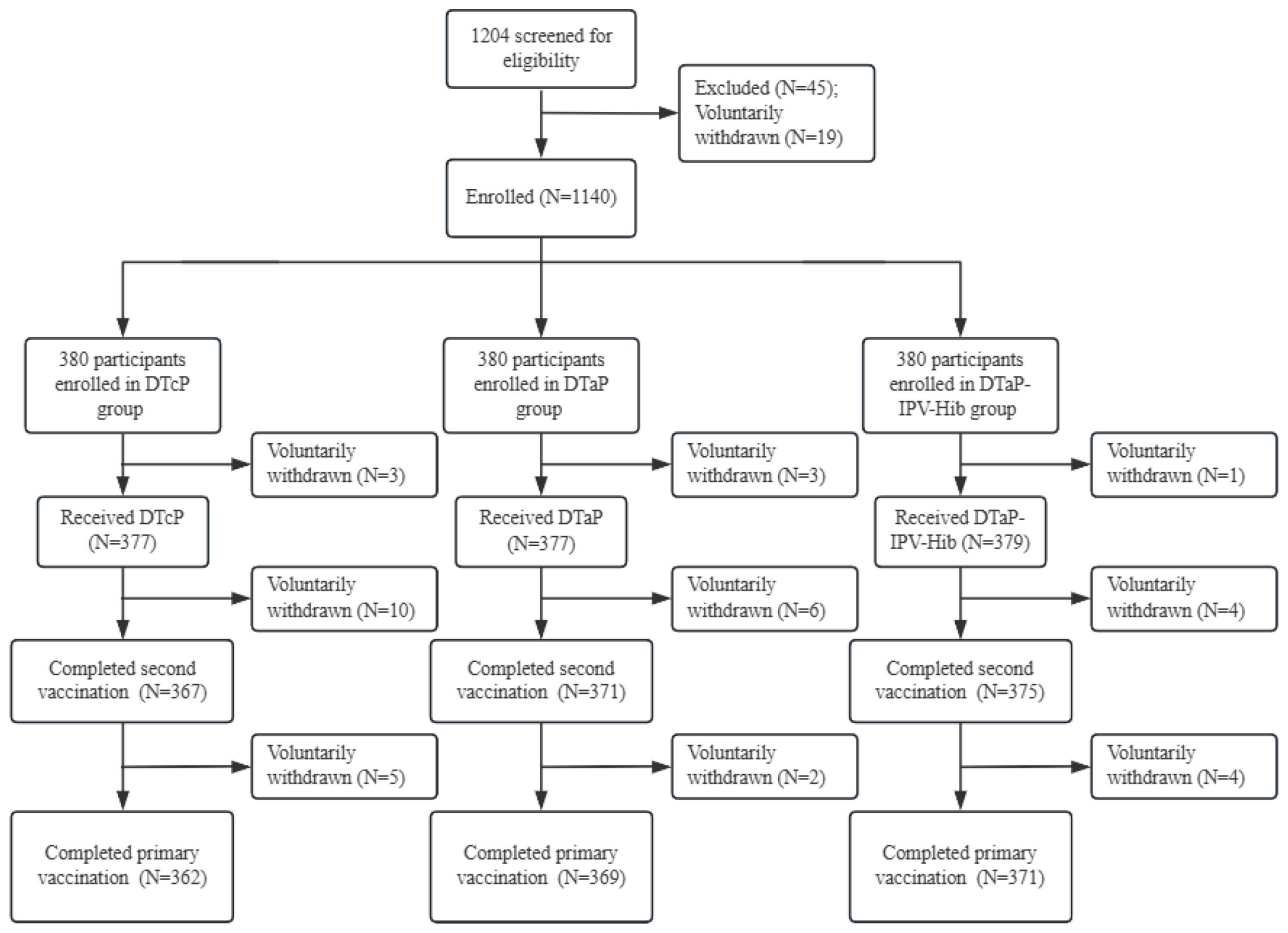
Flow diagram.

The study enrolled 1140 participants, with 760 randomly assigned to DTcP and DTaP in a 1:1 ratio and 380 assigned to DTaP-IPV-Hib (**Figure 1**). Baseline and demographic characteristics of participants were generally comparable across the three groups (**Table 1**). A statistically significant but clinically small difference was observed in mean age (*P* < 0.001).

**Table 1:** Baseline and demographic characteristics in participants.

Characteristics	DTcP (N = 377)	DTaP (N = 377)	DTaP-IPV-Hib (N = 379)	*P*
Gender, male (n)				
Male	208 (55.17%)	190 (50.40%)	196 (51.72%)	0.399
Female	169 (44.83%)	187 (49.60%)	183 (48.28%)	
Age (month)	3.39 (0.26)	3.38 (0.26)	3.29 (0.24)	**<0.001**
Height (cm)	63.36 (2.41)	63.38 (2.50)	63.12 (2.39)	0.430
Weight (kg)	7.02 (0.91)	6.99 (0.86)	6.88 (0.87)	0.102
Axillary temperature (°C)	36.69 (0.22)	36.68 (0.23)	36.72 (0.22)	0.057
Previous Medical History				
Yes	30 (7.96%)	19 (5.04%)	35 (9.23%)	0.079
No	347 (92.04%)	358 (94.96%)	344 (90.77%)	

Significant differences (*p* < 0.05) are shown in bold.

### Safety

Adverse reaction profiles are summarized in **Table 2**. The overall adverse reaction rate was significantly higher in the DTaP-IPV-Hib group (21.96%) compared to the DTcP group (17.99%, *P* = 0.019), while no significant difference was observed between DTcP and DTaP (*P* = 0.955). Local adverse reactions showed a similar pattern. Of note, swelling was less frequent in the DTaP group than in DTcP (3.13% vs. 4.88%, *P* = 0.035), whereas erythema showed the opposite trend (4.83% vs. 3.07%, *P* = 0.033). Among systemic reactions, fever was more common in the DTaP-IPV-Hib group (7.20% vs. 4.25%, *P* = 0.003). The incidence of cough was significantly lower in the DTcP group (2.98%) compared to the DTaP group (4.66%; *P* = 0.040). No vaccine-related serious adverse events occurred.

**Table 2:** Overview of adverse reactions within 30 days after primary vaccination.

Adverse reactions	DTcP (N = 1106)	DTaP (N = 1117)	DTaP-IPV-Hib (N = 1125)	P*	*P#*
Overall adverse reactions	199 (17.99%)	202 (18.08%)	247 (21.96%)	0.955	**0.019**
Local adverse reactions	71 (6.42%)	75 (6.71%)	101 (8.98%)	0.779	**0.024**
Systemic adverse reactions	122 (11.03%)	139 (12.44%)	154 (13.69%)	0.301	0.057
Swelling	54 (4.88%)	35 (3.13%)	64 (5.69%)	**0.035**	0.395
Erythema	34 (3.07%)	54 (4.83%)	51 (4.53%)	**0.033**	0.072
Induration	6 (0.54%)	7 (0.63%)	9 (0.80%)	0.795	0.457
Fever	47 (4.25%)	46 (4.12%)	81 (7.20%)	0.877	**0.003**
Irritability	11 (0.99%)	16 (1.43%)	9 (0.80%)	0.346	0.626
Diarrhea	29 (2.62%)	40 (3.58%)	38 (3.38%)	0.192	0.296
Vomit	8 (0.72%)	5 (0.45%)	7 (0.62%)	0.394	0.770
Nausea	0	1 (0.09%)	0	>0.999	–
Cough	33 (2.98%)	52 (4.66%)	47 (4.18%)	**0.040**	0.129
Drowsiness	1 (0.09%)	3 (0.27%)	4 (0.36%)	0.624	0.381

*Represented the comparison between DTcP and DTaP, ^#^represented the comparison between DTcP and DTaP-IPV-Hib.

Significant differences (*p* < 0.05) are shown in bold.

### Immunogenicity

Non-inferiority and superiority comparisons of seroconversion rates and GMCs are summarized in **Table 3**. For DTcP vs. DTaP, non-inferiority was established for all antigens (DT, TT, PT, and FHA), and superiority was demonstrated for anti-PT [difference 2.94% (95% CI: 0.49, 5.39); GMC ratio 1.71 (1.57, 1.86)] and anti-FHA [difference 83.87% (79.86, 87.89); GMC ratio 10.13 (9.23, 11.12)]. For DTcP vs. DTaP-IPV-Hib, non-inferiority was established for both PT and FHA, but superiority was not demonstrated.

**Table 3:** Non-inferiority/superiority comparison of seroconversion rates and GMC of antibodies between groups.

	Point estimate	Two-sided 95%CI	Non-inferiority cut-off	Established or not	Superiority cut-off	Established or not
Seroconversion rates						
DTcP VS DTaP						
DT	0	(-1.17, 1.13)	-0.1	Yes	0	No
TT	-0.01	(-0.85,0.83)	-0.1	Yes	0	No
PT	2.94	(0.49, 5.39)	-0.1	Yes	0	Yes
FHA	83.87	(79.86, 87.89)	-0.1	Yes	0	Yes
DTcP VS DTaP-IPV-Hib						
PT	0.51	(-1.31, 2.33)	-0.1	Yes	0	No
FHA	1.05	(-1.24, 3.34)	-0.1	Yes	0	No
GMC						
DTcP VS DTaP						
PT	1.71	(1.57, 1.86)	0.67	Yes	1	Yes
FHA	10.13	(9.23, 11.12)	0.67	Yes	1	Yes

For seroconversion rate, when the lower bound of the 95% CI on both sides of the point estimate >-0.1, the non-inferiority is established. The superiority is established when the lower bound of the two-sided 95% CI is >0. When the non-inferiority test is established, the superiority test is performed. For GMC, when the lower bound of the 95% CI on both sides of the point estimate ≥0.67, the non-inferiority is established. The superiority is established when the lower bound of the two-sided 95% CI is ≥1. When the non-inferiority test is established, the superiority test is performed.

The immunogenicity analysis 30 days post-primary immunization revealed distinct immune response profiles among the three vaccine groups, as detailed by seroconversion rates (**Table 4**). All vaccines induced near-universal seroconversion for DT and TT (>99%). For pertussis antigens, DTcP showed significantly higher seroconversion rates than DTaP for PT (98.77% vs. 95.83%, *P* = 0.020), FHA (98.16% vs. 14.29%, *P* < 0.001), and PRN (99.39% vs. 95.54%, *P* = 0.002). DTcP and DTaP-IPV-Hib showed comparable seroconversion rates for PT and FHA (both *P*>0.05).

**Table 4:** Seroconversion rates of anti-PT, anti-FHA, anti-PRN, anti-DT, and anti-TT antibodies 30 days after primary immunization.

Antibodies	DTcP (N = 326)	DTaP (N = 336)	DTaP-IPV-Hib (N = 346)	P*	*P#*
Anti-DT	326 (100%)	336 (100%)	345 (99.71%)	–	>0.999
Anti-TT	325 (99.69%)	335 (99.70%)	345 (99.71%)	>0.999	>0.999
Anti-PT	322 (98.77%)	322 (95.83%)	340 (98.27%)	**0.020**	0.823
Anti-FHA	320 (98.16%)	48 (14.29%)	336 (97.11%)	**<0.001**	0.372
Anti-PRN	324 (99.39%)	321 (95.54%)	0	**0.002**	**<0.001**

*Represented the comparison between DTcP and DTaP, ^#^represented the comparison between DTcP and DTaP-IPV-Hib.

Significant differences (*p* < 0.05) are shown in bold.

**Table 5** presented the GMCs for anti-pertussis (PT, FHA, PRN), anti-DT, and anti-TT antibodies before (Day 0) and 30 days after primary immunization. Baseline GMCs were low and comparable across all groups (all *P*>0.05). Post-vaccination, DTcP induced significantly higher anti-PT GMC than both DTaP (85.10 vs. 49.86, *P* < 0.001) and DTaP-IPV-Hib (85.10 vs. 74.25, *P* = 0.001). For anti-FHA, DTcP and DTaP-IPV-Hib both achieved high GMCs (108.28 and 121.04, respectively), while DTaP was markedly lower (10.69, *P* < 0.001). For anti-PRN (absent in DTaP-IPV-Hib), DTcP elicited a strong response (211.45) versus DTaP (62.83, *P* < 0.001). For DT and TT, all groups achieved protective levels despite some statistically significant differences.

**Table 5:** GMCs of anti-PT, anti-FHA, anti-PRN, anti-DT, and anti-TT antibodies before and 30 days after primary immunization.

Antibodies	DTcP (N = 326)	DTaP (N = 336)	DTaP-IPV-Hib (N = 346)	P*	*P#*
Day 0					
Anti-DT	0.00 (3.55)	0.00 (3.61)	0.00 (3.55)	0.812	0.160
Anti-TT	0.00 (3.34)	0.00 (3.50)	0.00 (3.71)	0.738	0.076
Anti-PT	0.49 (3.72)	0.53 (3.31)	0.51 (3.48)	0.699	0.412
Anti-FHA	2.13 (2.75)	2.05 (2.90)	2.15 (2.79)	0.627	0.710
Anti-PRN	0.44 (3.96)	0.51 (4.58)	0.47 (4.14)	0.151	0.304
Day 30					
Anti-DT	1.11 (1.92)	0.94 (2.07)	1.18 (2.04)	**0.002**	0.169
Anti-TT	3.28 (1.74)	2.34 (1.82)	4.23 (1.60)	**<0.001**	**<0.001**
Anti-PT	85.10 (1.77)	49.86 (1.70)	74.25 (1.70)	**<0.001**	**0.001**
Anti-FHA	108.28 (1.73)	10.69 (1.95)	121.04 (1.91)	**<0.001**	**0.001**
Anti-PRN	211.45 (1.87)	62.83 (1.88)	1.91 (1.12)	**<0.001**	**<0.001**

*Represented the comparison between DTcP and DTaP, ^#^represented the comparison between DTcP and DTaP-IPV-Hib. Standard deviations are shown in parentheses.

Significant differences (*p* < 0.05) are shown in bold.

The immunogenicity analysis 30 days post-primary immunization revealed distinct immune response profiles among the three vaccine groups, as detailed by GMIs (**Table 6**). DTcP induced significantly higher GMIs than DTaP for PT (173.47 vs. 94.62, *P* < 0.001), FHA (50.81 vs. 5.22, *P* < 0.001), and PRN (481.06 vs. 122.45, *P* < 0.001). DTcP and DTaP-IPV-Hib showed comparable GMIs for PT and FHA (both *P*>0.05). For DT, GMIs were similar across all groups. For TT, DTaP showed a lower GMI than DTcP (611.58 vs. 840.84, *P* = 0.006), while DTaP-IPV-Hib was comparable to DTcP.

**Table 6:** GMIs of anti-PT, anti-FHA, anti-PRN, anti-DT, and anti-TT antibodies 30 days after primary immunization.

Antibodies	DTcP (N = 326)	DTaP (N = 336)	DTaP-IPV-Hib (N = 346)	P*	*P#*
Anti-DT	479.47 (4.22)	415.87 (4.18)	449.99 (4.70)	0.144	0.682
Anti-TT	840.84 (4.01)	611.58 (4.14)	893.54 (4.52)	**0.006**	0.397
Anti-PT	173.47 (4.22)	94.62 (3.57)	146.40 (4.18)	**<0.001**	**0.046**
Anti-FHA	50.81 (3.40)	5.22 (3.36)	56.34 (3.86)	**<0.001**	0.300
Anti-PRN	481.06 (5.04)	122.45 (5.15)	4.04 (4.12)	**<0.001**	**<0.001**

*Represented the comparison between DTcP and DTaP, ^#^represented the comparison between DTcP and DTaP-IPV-Hib. Standard deviations are shown in parentheses.

Significant differences (*p* < 0.05) are shown in bold.

Seropositivity rates are presented in **Table 7**. Baseline seropositivity was low and comparable across groups (all *P*>0.05). Post-vaccination, all groups achieved 100% seropositivity for DT and TT. For PT, seropositivity was high in all groups, with DTcP (99.39%) significantly higher than DTaP (96.43%, *P* = 0.008) but comparable to DTaP-IPV-Hib (99.13%, *P*>0.999). For FHA, DTcP and DTaP-IPV-Hib achieved near-universal seropositivity (99.69% and 99.71%), while DTaP was markedly lower (16.37%, *P* < 0.001).

**Table 7:** Seropositivity rates of anti-PT, anti-FHA, anti-PRN, anti-DT, and anti-TT antibodies 30 days after primary immunization.

Antibodies	DTcP (N = 326)	DTaP (N = 336)	DTaP-IPV-Hib (N = 346)	P*	*P#*
Day 0					
Anti-DT	40 (12.27%)	47 (13.99%)	59 (17.05%)	0.513	0.080
Anti-TT	70 (21.47%)	81 (24.11%)	96 (27.75%)	0.419	0.059
Anti-PT	2 (0.61%)	2 (0.60%)	0	>0.999	0.235
Anti-FHA	4 (1.23%)	6 (1.79%)	2 (0.58%)	0.787	0.629
Anti-PRN	14 (4.29%)	24 (7.14%)	16 (4.62%)	0.115	0.836
Day 30					
Anti-DT	326 (100.00%)	336 (100.00%)	346 (100.00%)	–	–
Anti-TT	326 (100.00%)	336 (100.00%)	346 (100.00%)	–	–
Anti-PT	324 (99.39%)	324 (96.43%)	343 (99.13%)	**0.008**	>0.999
Anti-FHA	325 (99.69%)	55 (16.37%)	345 (99.71%)	**<0.001**	>0.999
Anti-PRN	326 (100.00%)	336 (100.00%)	1 (0.29%)	–	**<0.001**

*Represented the comparison between DTcP and DTaP, ^#^represented the comparison between DTcP and DTaP-IPV-Hib.

Significant differences (*p* < 0.05) are shown in bold.

## Discussion

This partially randomized, controlled trial demonstrated that the novel DTcP vaccine, which is produced using genetically engineered strains for separate antigen expression and purification, induces a robust immune response against *Bordetella pertussis* antigens following primary immunization in 3-month-old infants, while exhibiting a generally acceptable safety profile. The key findings revolved around the distinct immunogenicity and reactogenicity patterns observed among the three studied vaccines. Regarding safety, the DTcP vaccine demonstrated a comparable overall adverse reaction profile to the DTaP vaccine. However, it was associated with a significantly lower incidence of both overall and local adverse reactions compared to the DTaP-IPV-Hib combination vaccine. The higher reactogenicity of the combination vaccine is not unexpected, as increased antigenic load and the presence of multiple vaccine components often correlate with a higher incidence of local and systemic reactions (10). The distinct local reaction profile of the DTaP vaccine--less swelling but more erythema than DTcP--warrants further investigation but may relate to differences in the antigen-adjuvant formulation or the adsorption process. Fever was notably more common with the DTaP-IPV-Hib combination vaccine.

The immunogenicity data revealed critical differences, most notably between the DTcP and the DTaP vaccine. Our finding that the DTcP vaccine elicited significantly higher seroconversion rates and GMCs for both anti-PT (1.71-fold higher GMC) and anti-FHA (10.13-fold higher GMC) antibodies compared to the DTaP is a key observation. Most strikingly, while DTcP achieved a 98.16% seroconversion rate for anti-FHA antibodies, the co-purified DTaP vaccine elicited a profoundly deficient response, with a rate of only 14.29% and a correspondingly low GMC. This deficiency in the FHA response is a major weakness of the co-purified vaccine, as antibodies against FHA contribute to bacterial adhesion inhibition and broader immune protection (11). The superior immunogenicity of DTcP is likely attributable to its modern production platform, which utilizes genetically engineered strains for the separate expression, purification, and precise formulation of each antigen (PT, FHA, PRN), ensuring consistent antigenic composition and optimal immunogenic presentation. In contrast, the traditional co-purification process may yield a less defined antigenic mixture with variable composition, potentially compromising immunogenicity. A cross-sectional serological study compared the immune response of DTaP-IPV-Hib or co-purified DTaP vaccine found that the GMCs and seropositivity rates for anti-pertussis IgG antibodies, particularly anti-PT, were significantly higher in the component vaccine group than in the co-purified vaccine group. It also concluded that the component vaccine’s production process, which ensures antigenic consistency and reproducibility through separate purification and precise formulation, likely underpinned this immunogenicity advantage (8). Moreover, the dual-adjuvant system (aluminum hydroxide and aluminum phosphate) employed in DTcP may also have contributed to this enhanced immune response by creating a more favorable immunostimulatory environment (12). Additionally, all vaccines were highly immunogenic for the diphtheria and tetanus components, achieving 100% seropositivity rates. While the variations in GMIs for anti-TT were statistically significant, they are likely to be of limited clinical relevance, as they were far exceed the protective threshold (13). Besides, the DTcP vaccine also elicited consistently high seropositivity rates for all three key pertussis antigens: PT, FHA, and PRN. This comprehensive response is crucial, as antibodies against multiple antigens may contribute to broader protection against pertussis (14). In contrast, the control DTaP vaccine, while effective against PT and PRN, induced a strikingly poor anti-FHA response, with a seropositivity rate of only 16.37%. The DTaP-IPV-Hib combination vaccine generated strong anti-PT and anti-FHA responses, comparable to DTcP, while it does not contain PRN antigen (15). The absence of a PRN component is a significant limitation, as anti-PRN antibodies are thought to play a role in bacterial clearance and have been associated with clinical protection in some studies (16). This study also has limitations. The follow-up period for safety was limited to 30 days post-vaccination, and long-term persistence of antibodies and booster responses were not assessed here. Furthermore, another limitation is this study confinement to a single province in China, which may constrain the external validity and generalizability of the findings. Given China’s vast geographic, socioeconomic, and cultural diversity, results from one province may not be fully representative of the national population. Future multi-center studies spanning different regions are warranted to enhance the applicability of the conclusions.

In conclusion, the novel DTcP vaccine demonstrates a favorable immunogenicity and safety profile in infants. It induces a strong and balanced immune response against all three evaluated pertussis antigens, addressing the specific deficiencies observed in the control vaccines--namely, the poor anti-FHA response of the DTaP vaccine and the absent anti-PRN response of the DTaP-IPV-Hib vaccine. Given the unprecedented resurgence of pertussis in China and the evidence linking waning immunity to the vaccines currently in use, the findings from this trial provide a compelling scientific basis for the optimization and necessary upgrading of China’s EPI: transitioning from co-purified DTaP vaccines to more immunogenic, component-based aP vaccines. Future studies should focus on assessing the long-term antibody persistence and, crucially, the real-world vaccine effectiveness of this DTcP vaccine in preventing pertussis disease across age groups, especially in school-aged children. Confirming its impact on reducing transmission within this key demographic will be essential for optimizing China’s pertussis control strategy and protecting the most vulnerable infants.

## Data Availability

The raw data supporting the conclusions of this article will be made available by the authors, without undue reservation.
