# Supplementary material for: Immunogenicity and safety of primary three-dose series with diphtheria, tetanus and pertussis (acellular, three components) combined vaccine, adsorbed in 3 months infants
Source: Front Immunol. 2026 Jul 7;17:1874647. doi: 10.3389/fimmu.2026.1874647 (PMC13385028; doi:10.3389/fimmu.2026.1874647)
Supplement: Supplementary file 1 [file Supplementaryfile1.docx]

Exclusion Criteria:

**First Dose Exclusion Criteria:**

- Infants who have received vaccines containing pertussis, diphtheria, tetanus, or Haemophilus influenzae type b (Hib) components, or the 13-valent pneumococcal polysaccharide conjugate vaccine, or group A and group C meningococcal conjugate vaccine.
- Infants (DTaP-IPV-Hib Group) who have received IPV vaccine.
- Infants aged 3 months (90–119 days) who were born prematurely (delivered before the 37th week of pregnancy) or with low birth weight (<2500 g).
- Those with a history of abnormal labor, asphyxia resuscitation, or nervous system damage.
- Those who have previously had one of the following diseases: pertussis, diphtheria, or tetanus.
- Those who have had contact with individuals diagnosed with pertussis, diphtheria, or tetanus within their family in the past 30 days.
- Those with a history of allergy to vaccines or vaccine components, or who have experienced severe adverse reactions to vaccines, such as allergies, urticaria, dyspnea, or angioedema.
- Those with a history of epilepsy, convulsions, seizures, cerebral palsy, or a personal or family history of mental illness, or other progressive neurological diseases.
- Those diagnosed with congenital or acquired immunodeficiency, HIV infection, lymphoma, leukemia, systemic lupus erythematosus (SLE), juvenile rheumatoid arthritis (JRA), or other autoimmune diseases.
- Asplenia caused by any condition, or defects in splenic function.
- Known or suspected acute illness or severe chronic disease (including severe respiratory disease, severe cardiovascular disease, liver and kidney disease, severe skin disease, malignant tumors, etc.), or in the acute phase of a chronic disease.
- Physician-diagnosed coagulation abnormalities (e.g., coagulation factor deficiencies, clotting disorders, platelet abnormalities), or significant bruising or coagulation disorders.
- Receipt of immunosuppressants or immunomodulators, or continuous cytotoxic therapy for more than 10 days (excluding inhaled or topical steroids) within 3 months prior to receiving the investigational vaccine.
- Receipt of blood products (other than hepatitis B immune globulin) within 3 months prior to receiving the investigational vaccine.
- Receipt of other investigational drugs or investigational vaccines within 1 month prior to receiving the investigational vaccine.
- Planned participation or current participation in any other drug clinical study.
- Receipt of a live attenuated vaccine within 14 days prior to receiving the investigational vaccine, or other vaccines within 7 days prior.
- Axillary body temperature >37.0°C before vaccination.
- Any other factors that, in the investigator’s judgment, make the subject unsuitable for participation in the clinical trial.

**Exclusion Criteria for the Second and Third Doses:**

- Those who have had a severe allergic reaction after a previous dose of vaccination.
- Those who have experienced serious adverse reactions with a causal relationship to a previous dose of vaccination.
- Those who, after the first vaccination, are newly discovered or develop conditions that do not meet the initial dose inclusion criteria or meet the initial dose exclusion criteria; whether to continue participation shall be determined by the investigator.
- Other reasons for exclusion in the opinion of the investigator.
